# Reactivation of Chagas disease after autologous hematopoietic stem cell transplantation

**DOI:** 10.1590/0037-8682-0143-2020

**Published:** 2020-12-21

**Authors:** Claudia Marcela Chalela, Angela Maria Peña, Angela Maria Roa, David L. Reyes, Jennifer Paola Rueda, Luis Antonio Salazar, Manuel Rosales, Edgar David Gomez, Edgar Augusto Bernal, Claudia Lucia Sossa Melo

**Affiliations:** 1 Universidad Autónoma de Bucaramanga, Faculty of Health Sciences, Bucaramanga, Santander, Colombia.; 2 Clínica FOSCAL, Hematopoietic and Stem Cell Transplantation Unit, Floridablanca, Santander, Colombia.; 3 Clínica FOSCAL, Department of Internal Medicine, Floridablanca, Santander, Colombia.; 4 Clínica FOSCAL, Department of Infectious Diseases, Floridablanca, Santander, Colombia.

**Keywords:** Stem cell transplantation, Chagas disease, Trypanosoma cruzi

## Abstract

Chagas disease (CD) is a protozoan zoonosis caused by *Trypanosoma cruzi*. Reactivation of CD occurs via drug-induced immunosuppression before and during transplantation. Here, we report the case of a 62-year-old man diagnosed with classic Hodgkin lymphoma who received highly aggressive conditioning chemotherapy before undergoing stem cell transplantation (SCT). The patient tested positive for CD in pre-transplantation evaluation. The patient exhibited persistent fever and elevated C-reactive protein levels before and after SCT, and was treated with antibiotics. Micro-Strout test showed evidence of trypomastigotes and he was treated with benznidazole until tested negative. Post-transplantation seropositive patients should be screened for possible reactivation.

## INTRODUCTION

Chagas disease (CD), a protozoan zoonosis caused by *Trypanosoma cruzi (T. cruzi)*, is responsible for the highest morbidity and mortality related to parasitic infections in the Western Hemisphere, affecting approximately 7 million people worldwide^1^. CD is currently an endemic in Latin America with approximately 6 million infected individuals, and is a major public health concern in Central and South America, with an incidence rate of 28,000 cases per year^2^. In Colombia, approximately 437,960 people are infected with an estimated *T. cruzi* infection prevalence of 0.956 per 100 habitants^1^.

CD typically occurs in two stages, namely an acute stage and a chronic stage. An acute stage usually lasts for four to eight weeks and is characterized by high parasitemia and parasitic load in tissues. The symptoms include fever, hepatosplenomegaly, palpebral edema, and myocarditis. The chronic stage comprises two different distinguishable phases: a latent or indeterminate phase in which anti-*T. cruzi* antibodies are present, but no signs or symptoms of Chagas cardiomyopathy or gastrointestinal involvement are identified, followed by a determinate or clinical phase with prevalent cardiac, digestive, and cardiodigestive pathologies^3^. Reactivation of the disease occurs when the immune system of a chronically infected host is suppressed or compromised, thereby reducing their ability to control the infection and cause reappearance of acute symptoms. Reactivation of CD observed after transplantation may be related to immunosuppression post-transplantation^4^.

## CASE REPORT

**FIGURE 1:**
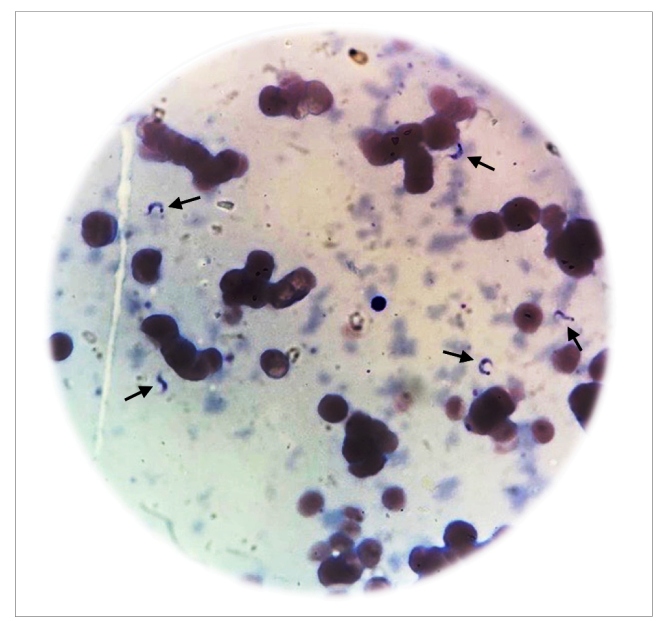
Micro-Strout test of the patient depicting trypomastigotes in peripheral blood sample.

A 62-year-old Colombian male diagnosed with classic Hodgkin lymphoma (HL) in October 2014 was treated with six cycles of ABVD (doxorubicin, bleomycin, vinblastine, and dacarbazine) until complete remission was achieved. After disease relapsed in January 2016, the next implementable management strategy was autologous hematopoietic stem cell transplantation (HSCT). In pre-transplant evaluation, the patient tested positive for CD upon serological testing, but no cardiac abnormalities were observed in his electrocardiogram or echocardiogram. The patient received salvage chemotherapy with three cycles of ESHAP (etoposide, methylprednisolone, cytarabine, and cisplatin) followed by a conditioning regimen of BEAM (carmustine, etoposide, cytarabine, and melphalan). In June 2016, three days prior to the transplantation, the patient was found to exhibit fever with elevated C-reactive protein levels, with no clinical evidence of any infection and negative blood cultures. Treatment with meropenem was initiated and three days later, on June 9, 2016, hematopoietic stem cells (11.14 × 10^6^/kg) were infused. On day 2 post-HSCT, after another episode of fever, vancomycin was added to the treatment therapy. Blood and urine cultures were negative, and imaging studies showed no evidence of infection. Despite empiric antibiotic treatment for 10 days, the patient presented persistent fever and elevated C-reactive protein levels. Management strategy included increased administration of meropenem, tigecycline, amikacin, and caspofungin. Myeloid engraftment occurred on day 12 post-HSCT. On day 17 post-HSCT, fever persisted and polymyxin B antibiotic was administered instead. Unfortunately, screening for reactivation of CD was not performed during the first two weeks post-transplantation. Thereafter, micro-Strout test was performed and showed evidence of trypomastigotes (Figure 1). No cutaneous alterations, such as nodules, papules, ulcers, or panniculitis were present. Neurological examination was also normal. Electrocardiography revealed a normal sinus rhythm and echocardiogram showed mild pleural effusion without any evidence of prolongation of isovolumic contraction and relaxation times, or dilation of the heart chambers. Chest CT scan was normal, and abdominal computed tomography showed mild splenomegaly, but no liver enlargement. Blood count, liver function tests, myelogram, and bone marrow biopsy results were also normal. Once the reactivation of CD was confirmed, therapy with nifurtimox at a dosage of 120 mg/day was initiated. Parasitological follow-up was performed with weekly micro-Strout tests, until the patient tested negative, which was observed after three weeks. Additionally, the patient presented primary graft failure requiring several platelet transfusions. Outpatient management with nifurtimox was continued for the patient until completing 60 days of treatment.

In October 2016, 54 days after completion of treatment with nifurtimox, the patient was admitted to the hospital for symptoms, such as general weakness, fatigue, intermittent fever, facial edema, and the patient tested positive in the micro-Strout test. Treatment was initiated with benznidazole at a dosage of 300 mg/day for 60 days, and the follow-up micro-Strout test was found to be negative three weeks later. Further parasitological controls were also negative. The patient has not presented present positive micro-Strout tests or symptoms suggestive of disease reactivation since then.

In January 2017, the patient presented with early relapse of HL, which was confirmed by positron emission tomography (PET scan) showing markedly hypermetabolic ganglion followed by positive axillary ganglion biopsy. Currently, he is on treatment for HL; however, there is no evidence of CD reactivation or complications associated with the disease.

## DISCUSSION

Reactivation of CD is usually associated with the state of immunosuppression. Several cases of reactivation following solid organ or hematopoietic stem cell transplantation, chronic use of immunosuppressants, or immunosuppression due to chronic diseases, such as HIV infection, have been reported. In Latin America, studies have reported reactivation rates in autologous and allogeneic HSCT cases between 17% and 40%^5^. Induced immunosuppression to prevent organ rejection in transplant patients causes a disruption in the immune system, which may result in reactivation of chronic CD^6^.

During the latent or undetermined phase of CD, the balance between T cell responses in the host maintains an equilibrium controlling the infection and destroys both trypomastigotes and intracellular amastigotes, thus limiting tissue damage and consequent symptoms^7^. CD4 lymphocytes are the predominant cells which induce protective immunity in Chagas infection^8^. Studies in both mice and humans have shown that type 1 T helper cells direct elicitation of antibody response, activation of other T cells, such as CD8 lymphocytes, and activation of phagocytes for parasite killing^8^ ^,^ ^9^. CD8 T cells can control infection by secreting cytokines that induce host-cell microbicidal and lytic activity^9^. These mechanisms are crucial for controlling parasitemia and infection. 

Highly aggressive chemotherapy, which is administered to transplantation patients prior to the procedure, induces severe immunosuppression which causes depletion of CD4 and CD8 lymphocytes. Deficiency of the cells that are essential for induction of immune response disrupts the balance to avoid progression from the indeterminate to the symptomatic chronic form in a chronically infected individual^10^. This imbalance leads to an increased parasite load and eventually causes the reactivation of CD.

Reactivation of CD can occur as a febrile syndrome that resembles acute graft failure. Clinical manifestations of disease reactivation in immunosuppressed patients also include myocarditis, cutaneous symptoms, such as subcutaneous nodules, papules, ulcers or panniculitis, anemia, jaundice, hepatitis, and meningoencephalitis^3^. In the present case, cellular exhaustion induced through long immunosuppressive therapies resulted in reactivation of latent CD that occurred as a febrile syndrome.

Although the diagnosis of chronic CD relies on serologic methods, immunosuppressed patients or patients who have received or who continue to receive immunosuppressive therapy may test negative in antibody detection analysis. To diagnose the reactivation of CD in immunocompromised patients, it is recommended to perform parasitological diagnosis using direct methods, such as the Strout test or microhematocrit and, if possible, quantitative PCR^11^. Although qualitative PCR is more sensitive than other parasitological methods, positive results of qualitative PCR as well as indirect parasitological methods (hemocultures) can also be considered in chronic disease in the absence of reactivation. Conversely, quantitative PCR can detect and differentiate low-level parasitemia observed in chronic CD from the high parasitemia levels observed during CD reactivation^12^ ^,^ ^13^. Therefore, diagnosis of CD reactivation should be based on quantitative PCR and/or microscopic examination of the buffy coat or fresh blood^3^ ^,^ ^12^.

In 2011, a research group with experts in Chagas disease published evidence-based recommendations for donor screening, follow-up testing, and treatment of organ recipients from infected donors (Chin-Hong *et al.*, 2011). Reactivation of CD can be treated with benznidazole or nifurtimox, as they are the only two drugs with proven efficacy against CD^14^. The consensus recommends benznidazole as the first-line treatment since it is better tolerated by the transplant recipients and has fewer drug interactions than nifurtimox. Nifurtimox is administered in patients who cannot tolerate benznidazole or if a previous therapy has failed^14^. Nifurtimox was administered as the first-line treatment to the patient discussed in this case report since benznidazole was not available in the hospital at the time of diagnosis.

In a prospective study conducted by Altclas *et al.* in Argentina, the effect of preemptive therapy in a cohort of 22 patients with CD who underwent bone marrow transplantation was evaluated, and the authors recommended treatment with benznidazole when subclinical or clinical manifestations of CD are present. They found that therapy with benznidazole for at least 30 days in reactivated CD recipients was the most appropriate strategy to clear parasitemia and to prevent new episodes of reactivation in HSCT patients with latent CD^6^. 

Additionally, a systematic review assessing the diagnosis and treatment of CD in the United States concluded that patients who underwent transplantation and presented reactivation of the infection were treated with standard doses of benznidazole for 30 to 180 days, which eventually resulted in the resolution of symptoms and reduced parasitemia^3^. 

Physicians have not reached a consensus on the use of prophylactic treatment to prevent CD reactivation in immunocompromised patients. Data on the efficacy of prophylactic treatment are insufficient, and the benefits of secondary prophylaxis have not been established thus far^3^. 

Current recommendations for monitoring of post-transplantation seropositive patients propose usage of systematic parasitological controls along with routine examination for detection of signs or symptoms of reactivation^6^. Laboratory testing includes basal pre-transplantation and weekly examination of blood smears by microscopy or quantitative molecular testing for two months, followed by bimonthly checks between two and six months post-transplantation, and annually thereafter^6^. Moreover, urgent parasitological screening should be performed in case of febrile syndrome or suspected reactivation of chronic CD. 

Our case report highlights the importance of considering the reactivation of chronic CD as a highly probable diagnosis in cases of febrile syndrome in a post-transplantation patient with a history of CD. Patients undergoing HSCT are at an increased risk of CD reactivation since their immune system is compromised, which results in the suppression of the pathways involved in controlling the infection. It is crucial to screen patients for CD who opt for stem cell transplantation in endemic countries, and to continue close systematic parasitological testing in post-transplantation patients with latent disease. Moreover, further research is warranted to study the implications of prophylactic treatment in bone marrow transplant recipients with latent CD.
